# Epidemic of parvovirus B19 and disease severity in pregnant people, Denmark, January to March 2024

**DOI:** 10.2807/1560-7917.ES.2024.29.24.2400299

**Published:** 2024-06-13

**Authors:** Anne Christine Nordholm, Frederik Trier Møller, Signe Fischer Ravn, Lotte Flink Sørensen, Anja Moltke-Prehn, Jacob Elskær Mollerup, Tjede Funk, Lene Sperling, Ulisa Jeyaratnam, Kristina Træholt Franck, Karina Hjort-Pedersen, Christina Hjørnet Kamper, Rikke Thoft Nielsen, Pikka Jokelainen, Maria Wessman

**Affiliations:** 1 Department of Infectious Disease Epidemiology and Prevention, Statens Serum Institut, Copenhagen, Denmark; 2 Department of Data Integration and Analysis, Statens Serum Institut, Copenhagen, Denmark; 3 Department of Gynaecology and Obstetrics, Odense University Hospital, Odense, Denmark; 4 Department of Virus and Microbiological Special Diagnostics, Statens Serum Institut, Copenhagen, Denmark; 5 Department of Gynaecology and Obstetrics, Aarhus University Hospital, Aarhus, Denmark; 6 Infectious Disease Preparedness, Statens Serum Institut, Copenhagen, Denmark

**Keywords:** Fifth disease, erythema infectiosum, pregnancy, epidemiology, surveillance, public health, parvovirus B19

## Abstract

We report an epidemic of parvovirus B19 infections in Denmark during the first quarter of 2024, with a peak incidence 3.5 times higher than during the most recent epidemic in 2017. In total, 20.1% (130/648) of laboratory-confirmed cases were pregnant. Severe adverse outcomes were observed among 12.3% (16/130) of pregnant people and included foetal anaemia, foetal hydrops and miscarriage. Parvovirus B19 infection is not systematically monitored, but a national laboratory-based surveillance system is currently being established in Denmark.

Parvovirus B19 infection, a common childhood infection, is an important concern for pregnant people in Europe, where up to 40% may be susceptible to the infection [1,2]. Parvovirus B19 usually causes mild disease, but it poses a risk to seronegative pregnant people, potentially resulting in severe outcomes such as anaemia, hydrops fetalis, foetal death or miscarriage [1,2]. In March 2024, clinicians in Denmark notified an increased number of hospitalisations and complications in pregnant people with parvovirus infection to the national public health institute, Statens Serum Institut (SSI), and a register-based study was initiated to investigate epidemiological trends and disease severity.

## Surveillance and diagnostics of parvovirus B19 infections

In Denmark, surveillance of parvovirus B19 infections is currently lacking, with no standardised testing protocols and limited understanding of the overall disease burden, mirroring the situation in other European countries [2]. As the majority of infections are self-limiting, often mild, and mainly affect paediatric populations, most cases are not laboratory-confirmed. On clinical suspicion, diagnosis typically involves serological testing with ELISA (IgM) and/or PCR, conducted at local clinical microbiology facilities or the Statens Serum Institut (SSI). Results are subsequently registered in the Danish Microbiology Database (MiBa).

We included data from 1 January to 30 April 2024 and compared with previous years. Using the unique Danish personal identification number (CPR number), information was linked on laboratory-confirmed parvovirus B19 infections from MiBa with demographic data from the Civil Registration System, hospitalisation records and information on severe outcomes among foetuses from the Danish National Patient Registry and information on pregnancies from the National Health Insurance Service Registry and the Danish National Patient Registry. Parvovirus B19 infections are endemic in Denmark with an annual seroconversion rate of 1.5% [3] and with epidemics occurring around every 3–4 years, typically during the spring months [4], when the annual seroconversion rate is 13% [3].

## The epidemiology of laboratory-confirmed parvovirus B19 infections

**Figure 1:**
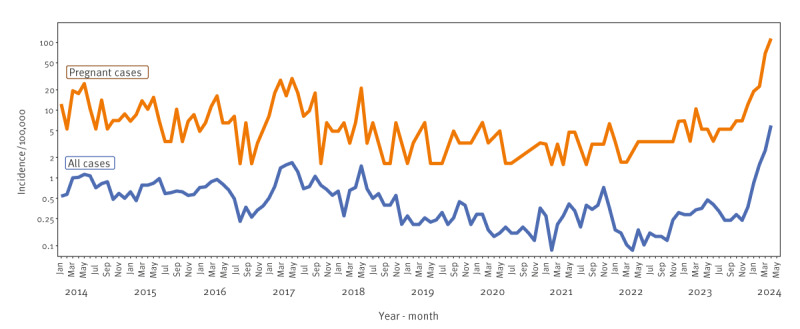
Incidence of parvovirus B19 infections and hospital admissions among pregnant cases and all confirmed cases, by year and month, Denmark, January 2014–April 2024 (n = 3,989)

From 1 January to 30 April 2024, 648 cases of parvovirus B19 infections were notified in Denmark among children and adults. The number of laboratory-confirmed cases is probably much lower than the true number of parvovirus B19 infections in Denmark. Test practices are most likely biased as healthy individuals are rarely tested for parvovirus B19 infection, whereas pregnant people and individuals with haematological disorders or immunodeficiencies are more frequently tested. The incidence peaked at 5.93 per 100,000 population in April, which is 3.5 times higher than in the most recent epidemic (March–June 2017) with a peak incidence of 1.68 per 100,000 population in May and a total of 339 cases. From 2014 to 2023, the average interepidemic incidence in Denmark was 0.45 per 100,000 population, excluding the epidemic months of 2017 (Figure 1). During the current epidemic, the confirmed cases were aged 0–89 years, of whom 80.7% were women and 376/648 (58.0%) of them were aged 30–59 years.

Incidence axis is log10-scaled.

The number of all cases was calculated using a background population of all Danish residents on 1 January of the year in question.

The number of pregnant cases was calculated using a background population of the number of births in Denmark per year.

The interval between this and the previous epidemic is longer than the usual 3–4 years, and there are more cases than during previous epidemics, which might be attributed to the non-pharmaceutical interventions introduced to avert COVID-19 transmission in society. In January 2024, the number of laboratory-confirmed parvovirus B19 infections increased remarkably (Figure 1). The positivity rate for the current epidemic in 2024 (January–April) is 7.0% (648/9,228) and 1.5 times higher than in the last epidemic of 2017 (4.1%, 339/8,336) and considerably higher than during the interepidemic period (1.7%, 3,002/180,918).

## Parvovirus B19 infections during pregnancy

In the current epidemic, 20.1% (n = 130) of the 648 laboratory-confirmed cases were pregnant (Table). In total, 12.3% (16/130) of these pregnant people experienced adverse severe outcomes (Table), compared with 5 of 56 (8.9%) during the 2017 epidemic and 38 (10.0%) of 379 during the interepidemic period. However, the proportion of cases identified among pregnant people is subject to test practices as relatively more pregnant people are tested to screen and treat foetal complications.

**Table:** Pregnant people with laboratory-confirmed parvovirus B19 infection and complications, Denmark, January 2014–April 2024 (n = 565)

Characteristics	Interepidemic 2014–2023^a^			Epidemic 2017			Epidemic 2024		
	n	%	Incidence^b^	n	%	Incidence^b^	n	%	Incidence^b^
Pregnant people	379		5.4	56		22.8	130		56.1
Pregnant people with severe adverse outcome	38	10	63.4	5	8.9	24.4	16	12.3	82.8
Type of severe outcome^c^									
Anaemia	11	2.9	1.8	2	3.6	9.8	8	6.2	41.4
Fetal hydrops	4	1.1	0.7	1	1.8	4.9	1	0.8	5.2
Miscarriage	26	6.9	4.4	2	3.6	9.8	5	3.8	25.9
Fetal transfusion	2	0.5	0.3	3	5.4	14.7	9	6.9	46.6

^a^ The epidemic months in 2017 (March–June) are not included.

^b^ Incidence is calculated as an average per month for the epidemic periods, which in 2017 was March–June and in 2024 January–April 2024.

^c^ Individuals may have experienced multiple outcomes and two pregnant individuals had foetal transfusions without a diagnosis of either anaemia, hydrops fetalis or miscarriage in the national inpatient registry.

**Figure 2:**
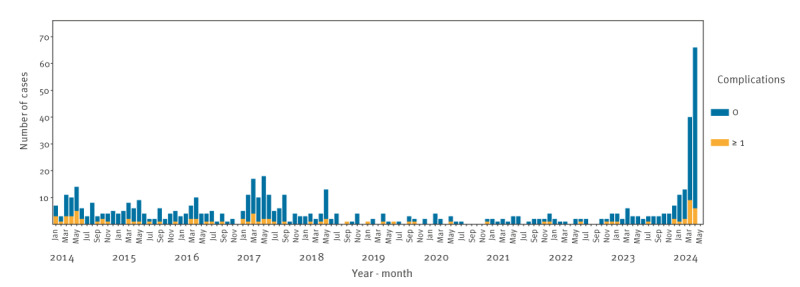
Pregnant people with laboratory-confirmed parvovirus B19 infection and complications, by month, Denmark, January 2014–April 2024 (n = 565)

Overall, the number of pregnant people experiencing severe adverse outcomes is generally low in Denmark, but the proportion is higher during the current epidemic compared with previous years (Figure 2). The proportion of miscarriages during the 2017 epidemic and the current epidemic are similar (3.6% vs 3.8% of cases) (Table).

Among all miscarriages registered from January to April 2024, 0.1% (5/4,429) were associated with parvovirus B19 infection, which is slightly higher than during the epidemic in 2017 (0.03%, 2/5,639) and expectedly higher than during the interepidemic periods (0.01%, 26/180,670). During the current epidemic, more foetal transfusions have been performed than during the previous epidemic in 2017 (9 vs 3) (Table).

## Discussion

Since January 2024, Denmark has experienced an epidemic of parvovirus B19 infections, with 648 notified cases as of 30 April, of which 130 were pregnant people. The peak incidence and proportion of positive test results were the highest reported in the past 10 years and were 3.5 times and 1.5 times higher than during the 2017 epidemic, respectively. At least one severe adverse outcome was observed among 12.3% (16/130) of pregnant individuals and included anaemia, foetal hydrops, foetal transfusions and miscarriages.

Parvovirus B19 targets erythroid progenitor cells in the bone marrow and in the foetal liver, which can induce anaemia [5]. In our study, we could not determine the proportion of foetuses being infected, but the risk of foetal loss is comparable to other studies (3.8%, 5/130) [6,7]. Foetal hydrops is rare in Denmark; only one case has been identified in 2024 corresponding to 0.8%, whereas other studies have found foetal hydrops in 4–13% of pregnant people infected with parvovirus B19 before the week 20 of gestation [2,6,7]. Adverse severe outcomes have mainly been demonstrated in pregnant people infected during the first or second trimester, whereas infection acquired later in pregnancy has less frequently been associated with increased risk for the foetus [8]. On suspicion of parvovirus B19 infection in pregnant people, serial ultrasounds with Doppler are conducted to discover any signs of foetal anaemia or hydrops [9]. There is no treatment or vaccine against the infection, however, intrauterine transfusion to treat anaemia is often successful [9,10]. During the current epidemic, nine individuals had intrauterine transfusions performed, more than during the 2017 epidemic when three were performed.

Pregnant people are not routinely screened for parvovirus B19 antibodies in Denmark. A previous study has demonstrated that 65% of pregnant people in Denmark had evidence of previous parvovirus B19 infection [3]. As this infection induces life-long immunity, the remaining 35% are susceptible to infection. The risk of acquiring parvovirus infection has been associated with the level of contact to children [11], and the risk of transplacental infection during pregnancy has been estimated to be around 30% [12]. The Danish Health Authority does not recommend absence from work for pregnant people working with children during parvovirus B19 outbreaks, unless extraordinary circumstances support such measures, e.g. a pregnant individual suffering from haematologic or blood disorders [13,14]. Infection rarely causes hospitalisations and of notice, there have been no deaths among children with parvovirus infection registered during the current epidemic, as opposed to the five deaths reported in France by Santé publique [15]. According to the European Centre for Disease Prevention and Control, several countries have reported an increase in the number of parvovirus cases per country [16]. Raised awareness of increased occurrence of the disease is important, to facilitate diagnosis of pregnant people with infection [16].

## Conclusion

Since January 2024, there is an ongoing parvovirus B19 epidemic in Denmark, the largest seen in the past 10 years. Among pregnant people with parvovirus infection, severe adverse outcomes were observed among 12.3% of which 3.8% had a miscarriage. Diagnostics of parvovirus B19 infection among pregnant people is important, because foetal anaemia caused by the virus can be treated with intrauterine transfusions. Overall, among all miscarriages in 2024 in the country, parvovirus B19 infections are only rarely associated with miscarriages (0.1%, 5/4,429). National laboratory-based surveillance of parvovirus B19 infections is currently being implemented.
